# Evaluation of a Low-Temperature Immersion Immunization Strategy for the Infectious Spleen and Kidney Necrosis Virus *orf037l* Gene-Deleted Attenuated Vaccine

**DOI:** 10.3390/vaccines12101170

**Published:** 2024-10-14

**Authors:** Weiqiang Pan, Jiajie Fu, Ruoyun Zeng, Mingcong Liang, Yanlin You, Zhipeng Zhan, Zhoutao Lu, Shaoping Weng, Changjun Guo, Jianguo He

**Affiliations:** School of Marine Sciences, State Key Laboratory for Biocontrol & Southern Laboratory of Ocean Science and Engineering (Guangdong, Zhuhai), Guangdong Province Key Laboratory of Aquatic Economic Animals/Guangdong Provincial Observation and Research Station for Marine Ranching of the Lingdingyang Bay, Sun Yat-sen University, 135 Xingang Road West, Guangzhou 510275, China; panwq5@mail2.sysu.edu.cn (W.P.); fujj3@mail2.sysu.edu.cn (J.F.); zhanzhp3@mail2.sysu.edu.cn (Z.Z.);

**Keywords:** vaccine, low-temperature immunization, *Siniperca chuatsi*, ISKNV, virulence, aquaculture

## Abstract

Background: Infectious spleen and kidney necrosis virus (ISKNV) poses a significant threat to aquaculture sustainability, particularly affecting mandarin fish (*Siniperca chuatsi*) and causing significant economic losses. Methods: To address this challenge, this study developed an ISKNV Δ*orf037l* vaccine strain, where the *orf037l* gene was knocked out. Infection assays conducted at 28 °C showed that the knocking out the *orf037l* gene decreased the virulence of ISKNV and reduced lethality against mandarin fish by 26.7% compared to wild-type ISKNV. To further diminish residual virulence, the effect of low-temperature (22 °C) immersion immunization was evaluated. Results: The results indicate that low temperature significantly diminished the virulence of the Δ*orf037l* vaccine strain, elevating the survival rate of mandarin fish to 90%. Furthermore, the vaccine strain effectively triggered the expression of crucial immune-related genes, such as *IFN-h*, *IL-1*, *IκB*, *Mx*, *TNF-α*, and *Viperin*, while inducing the production of specific neutralizing antibodies. Low-temperature immersion with Δ*orf037l* achieved a high relative percentage of survival of 92.6% (*n* = 30) in mandarin fish, suggesting the potential of Δ*orf037l* as a promising immersion vaccine candidate. Conclusions: These findings contribute to advancing fish immersion vaccine development and demonstrate the importance and broad applicability of temperature optimization strategies in vaccine development. Our work carries profound implications for both the theoretical understanding and practical application in aquaculture disease control.

## 1. Introduction

Infectious spleen and kidney necrosis virus (ISKNV), which belongs to the genus *Megalocytivirus* in the family *Iridoviridae*, is a double-stranded DNA-containing virus [1]. According to the latest ICTV report, the genus *Megalocytivirus* was classified into two species: *Megalocytivirus lates1* and *Megalocytivirus pagrus1* [2,3]; the latter is further categorized into three subtypes, namely, red seabream iridovirus (RSIV), ISKNV, and turbot reddish body iridovirus [4]. ISKNV infections have been observed in over 60 freshwater and marine fish species, with the mandarin fish (*Siniperca chuatsi*) being a primary host [1,5,6,7,8,9,10,11]. Recently, infectious spleen and kidney necrosis disease (ISKND) caused by ISKNV infection has caused significant economic losses in Asia [12,13,14,15,16,17], particularly in China, where it has led to high mortality rates and substantial economic losses in mandarin fish aquaculture [5,18]. However, there are no effective therapeutic approaches available for the treatment of ISKND [19,20]. Therefore, the aquaculture industry has focused on prevention measure to address ISKND, with vaccines being the most promising tools [21]. In Asia, commercial vaccines are available for the control of ISKND in sea bass and Nile tilapia [22,23]. In China, an inactivated ISKNV vaccine for injection immunization of mandarin fish has been commercially developed [4]; however, immersion vaccines and live-attenuated vaccines have yet to be commercialized.

Fish immunization has been practiced for over 50 years and is widely recognized as an effective method for preventing various bacterial and viral diseases. Vaccination efforts contribute to the environmental, social, and economic sustainability of global aquaculture [24,25]. Since the 1980s, significant progress has been made in the development of aquatic vaccines, with the number of commercially available fish vaccines against major bacterial and viral diseases continuously increasing [26,27,28]. By 2020, over 140 aquatic vaccines had been globally approved, including whole inactivated vaccines, peptide subunit vaccines, recombinant protein vaccines, nucleic acid vaccines, and live-attenuated vaccines [29]. Notably, intraperitoneal injection remains the primary delivery method for most commercial vaccines [30], yet this route may not be the optimal for large-scale vaccinations, with its effectiveness frequently influenced by various factors such as animal species, immune status, production cycle, environmental conditions, nutrition, and cost-effectiveness [21]. In contrast, immersion immunization offers advantages, allowing antigens to enter the body through multiple fish tissues and induce systemic immune responses, with minimal mechanical damage and stress to the fish [31,32]. This method facilitates the development of mass vaccination, reduces labor intensity, and shortens the immunization duration, making it suitable for fry and smaller fish [21]. Gene-deleted live-attenuated vaccines, created by removing virulence genes from the viral genome, mimic natural infection and trigger robust antibody responses, making them ideal for immersion immunization [24,33]. Several successful live-attenuated ISKNV vaccines, including Δ*orf022l*, Δ*orf074l*, Δ*orf069l*, and Δ*orf103r/tk* gene-deletion vaccines, have been developed. All these vaccines can be administered via immersion or injection, yet their residual virulence and protective effects vary. [34,35,36,37]. This phenomenon can be attributed to the differences in the knocked-out genes, as well as the varying degrees of their contributions to viral virulence. The selection of virulence-related genes is crucial for the development of live-attenuated vaccines, as it directly impacts their efficacy. Consequently, identifying and characterizing new virulence-related genes can accelerate the development of live-attenuated vaccines.

Temperature, a primary abiotic factor, regulates and influences the development, physiology, and disease outbreak in aquatic animals throughout all stages of aquaculture [38,39]. Model predictions suggest that a 1 °C temperature increases can lead to a 2.82–4.12% increase in mortality among warm-water and temperate organisms and a 3.87–6.00% increase among temperate organisms [40]. Temperature also affects the ISKND in mandarin fish, with temperatures above 25 °C resulting in 100% mortality and temperatures below 20 °C being non-pathogenic [1]. Studies have confirmed that some viruses, such as nervous necrosis virus [41], viral hemorrhagic septicemia virus [42], and red sea bream iridovirus [43], can confer immune protection to the host when infected at non-lethal temperatures. Recently, our laboratory reported that temperature optimization can reduce the virulence of live-attenuated virus vaccines, thereby enhancing their safety and efficacy [34]. Hence, optimizing the environmental temperature is a crucial factor to consider during the development of vaccines targeting fish viruses.

ISKNV *orf037l* encodes a protein with an unknown function. However, this protein is also identified in other members of the *megalocytiviruses* such as rock bream iridovirus [44] and orange-spotted grouper iridovirus (OSGIV) [45]. Its role as a virulence-related gene remains unknown. In this study, an ISKNV strain with the *orf037l* gene deleted (Δ*orf037l*) was constructed and a low-temperature immersion immunization protocol was designed through temperature optimization, rendering it a promising candidate as an immersion vaccine for ISKNV. Our work aims to provide new insights into the virulent viral genes of the iridovirus and further validate the importance of water temperature in immersion immunization.

## 2. Materials and Methods

### 2.1. Animals, Cells, and Viruses

Mandarin fish (*S. chuatsi*), confirmed to be ISKNV-free through polymerase chain reaction (PCR) testing, were supplied by a farm in Foshan, Guangdong, China, with an average weight of 30 ± 5 g. All fish were divided into groups of 30 and temporarily kept in a recirculating aquaculture system in the laboratory, with water temperature maintained at 27 ± 0.5 °C and the fish fed once a day until the start of the experiment. All animal experimentation adhered strictly to the regulations set by Guangdong Province, China, and was approved by the Ethics Committee of Sun Yat-sen University (approval no. 2020072301). The mandarin fish fry (MFF-1) cells were cultivated in Dulbecco’s modified Eagle’s medium (DMEM; Gibco, Grand Island, NY, USA) supplemented with 10% fetal bovine serum (HyClone, Logan, UT, USA) and maintained in a humidified environment with 5% CO_2_ [46]. The wild-type (WT) ISKNV strain (OP896201.1) was isolated from mandarin fish and preserved in our laboratory [34].

### 2.2. Construction of the ISKNV ∆orf037l Recombinant Transfer Vector

The pUC19-*rfp*-*puro* vector was engineered by sequentially inserting the red fluorescent protein (*rfp*) gene and the puromycin resistance (*puro*) gene, controlled by the CMV promoter, into the pUC19 vector, as described in a previous study [35]. The left and right genome fragments of approximately 1000 bp that flank the ISKNV *orf037l* gene were amplified through PCR by using the primers listed in Table 1. Subsequently, the left and right fragments of ISKNV *orf037l* were attached to both sides of the reporter gene on the vector using the restriction enzymes EcoR I/BamH I and Kpn I/Hind III to form the ISKNV *orf037l* recombinant transfer vector, designated as pUC19-Δ*orf037l*.

### 2.3. Construction of ISKNV Δorf037l Deletion Recombinant Viral Strain

Cells were seeded in six-well cell culture plates, and the pUC19-Δ*orf037l* vector was transfected using FuGENE^®^ HD transfection reagent (Promega, Madison, WI, USA). Following a 24 h post-transfection period, the cells were infected with WT ISKNV at a multiplicity of infection (MOI) of 0.01 to generate recombinant viruses. Healthy cells infected with these recombinant viruses and puromycin (at a 2 μg/mL concentration) were used to eliminate cells uninfected by the recombinant viruses. Red fluorescent plaques formed by the recombinant viruses were then picked out using an inverted fluorescence microscope. This process was repeated thrice to obtain the purified recombinant viral strain, termed Δ*orf037l*. Complete substitution of *orf037l* with the reporter genes was verified through PCR using *orf037l* outer primers (Table 1).

### 2.4. Assessment of the Expression of Genes Flanking orf037l

Cells were infected with Δ*orf037l* or WT ISKNV at an MOI of 0.1. After 72 h of infection, RNA samples were extracted from the cells and converted into cDNA. The expression levels of *orf035l*, *orf036r*, *orf038l*, and *orf039r* were quantified using real-time quantitative PCR (RT-qPCR) methods. RT–qPCR assays were conducted using TB Green^®^ Premix Ex Taq™ II (TAKARA, Kusatsu, Japan) with a Roche LightCycler^®^ 480 System, while *S. chuatsi β-actin* was used as the reference gene. The reaction protocols were as follows: 50 °C for 2 min and 95 °C for 10 min, followed by 40 cycles of 95 °C for 15 s, 60 °C for 15 s, and 72 °C for 15 s. The primers employed are listed in Table 1. Experiments were conducted in triplicate for each sample.

### 2.5. Assessment of Δorf037l Virulence

Fish were exposed to Δ*orf037l* or WT ISKNV via immersion at a concentration determined by qPCR to be 1 × 10^5^ copies/mL. In the control group, fish were immersed in water containing an equivalent volume of DMEM. Each treatment group consisted of 30 mandarin fish with an average weight of 30 g, which are immersed in separate aquariums at 28 °C for 4 h, and subsequently maintained in a 50 L glass tank equipped with an air-pumped circulating water system, where they are fed daily. After a 30-day feeding period, relative percentage of survival (RPS) were calculated.

### 2.6. Immunization Procedure with Δorf037l

Fish were immersed in Δ*orf037l* or WT ISKNV at a concentration of 1 × 10^5^ copies/mL at 22 °C. Fish immersed in water with an equal volume of DMEM served as the blank control group. Thirty fish from each experimental group were immersed for 4 h, maintained at 22 °C for 30 days, and then the water temperature was adjusted to 28 °C for 10 days. The survival rate was monitored and calculated. At 40 days post-immersion, fish from the Δ*orf037l* or control group were challenged with 1 × 10^6^ copies/mL of WT ISKNV via immersion, and their survival rates were tracked 21 days following infection.

### 2.7. Histopathological Sections and Hematoxylin–Eosin (H&E) Staining

Fish were immersed in Δ*orf037l*, WT ISKNV, or DMEM at a concentration of 1 × 10^5^ copies/mL and maintained at either 28 °C or 22 °C. Spleen samples were collected and sectioned at 9, 12, and 15 days post-immersion. The sections were stained with H&E and subsequently examined under a microscope.

### 2.8. Expression Levels of Immune-Related Genes in Mandarin Fish Induced by Δorf037l

To determine the expression levels of the immune-related genes (*IFN-h*, *IL-1*, *IκB*, *Mx*, *TNF-α,* and *Viperin*) following infection, fish were exposed to Δ*orf037l* at a concentration of 1 × 10^5^ copies/mL by immersion at either 28 °C or 22 °C (with 30 fish per group). Spleen samples were collected at 0, 3, 6, 9, 12, and 15 days post-infection, with three fish samples per day from each group. RNA was extracted from these samples using an SV Total RNA Isolation System (Promega, Madison, Wisconsin, USA) and subsequently reverse-transcribed into cDNA using *Evo M-MLV* RT Premix (Accurate Biology, Changsha, China), following the manufacturer’s protocol. The cDNA samples were then quantified for immune-related gene levels via RT-qPCR, as previously described. *IFN-h*, *IL-1*, *IκB*, *Mx*, *TNF-α*, and *Viperin* relating to innate immunity were detected. Mandarin fish *β-actin* served as the reference gene, and the primers used are listed in Table 1. The expression levels of the target genes were calculated using the 2^−ΔΔ^*^Ct^* quantification method.

### 2.9. Neutralization Assay

Serum samples were collected from mandarin fish that were immersed in Δ*orf037l* at a concentration of 1 × 10^5^ copies/mL or DMEM at 28 °C or 22 °C, 12 days after immunization. These samples were incubated with an ISKNV suspension (2.2 × 10^8^ copies/mL) for 12 h at 4 °C, either undiluted or diluted at ratios of 1:1, 1:5, 1:10, or 1:20 in DMEM. The incubated mixtures were then used to infect MFF-1 cells at an MOI of 0.01. For RT-qPCR assay, RNA was extracted from the cells 72 h post-infection and reverse-transcribed to cDNA. The levels of ISKNV major capsid protein (*mcp*) gene in the cDNA were quantified as a reference for RT-qPCR method. For Western blot analysis, cell samples at 72 h post-infection were prepared using Omni-Easy™ Protein Sample Loading Buffer (Epizyme, Shanghai, China) and boiled for Western blot analysis to determine the viral protein in MFF-1 cells. Equal quantities of protein samples were separated using SDS-PAGE and subsequently transferred to a 0.45 µm PVDF membrane (Millipore, Burlington, MA, USA). A structural protein of ISKNV, *orf101l*, which is one of the most abundant proteins in ISKNV, was used as a representative of viral load, with glyceraldehyde-3-phosphate dehydrogenase (GAPDH) as a reference for protein. In the microneutralization (MN) assay, serum samples underwent serial dilution with DMEM in two steps, as follows: The diluted serum was mixed with one hundred 50% tissue culture infective doses (TCID_50_) of ISKNV suspension at a 1:1 ratio in 96-well plates and incubated overnight at 4 °C. Following this, 2 × 10^4^ MFF-1 cells were added to the serum–virus mixture and incubated for 5 days at 27 °C in a 5% CO_2_ incubator. The wells with the enlarged cells were recorded, and neutralization titers were expressed as MN_50_, representing the reciprocal of the serum dilution neutralizing 50% of the test virus dose [47].

### 2.10. Determination of ISKNV-Specific IgM via Enzyme-Linked Immunosorbent Assay (ELISA)

Serum samples were collected from fish 12 days after immersion in Δ*orf037l* or DMEM at a dose of 1 × 10^5^ copies/mL. The levels of ISKNV-specific IgM in these samples were measured using ELISA, as previously described [35]. In brief, purified ISKNV was coated onto an ELISA plate (Nunc MaxiSorp, BioLegend, San Diego, CA, USA) in carbonate–bicarbonate buffer at pH 9.8 and incubated overnight at 4 °C. The coated plates were then blocked with 5% goat serum (Boster, Zhongshan City, China) in phosphate-buffered saline (PBS) containing 0.05% Tween-20 (PBST) overnight at 4 °C. Subsequently, serum samples were diluted 1:100 in 5% PBST, added to the plates, and incubated for 2 h at room temperature. After washing with PBST, plates were sequentially hybridized with mandarin fish IgM-specific antibody and horseradish peroxidase-labeled goat anti-mouse antibody to detect ISKNV-specific antibodies. Absorbance (optical density) was measured at 405 nm using Synergy Neo2 Hybrid Multimode Reader (Biotek, Winooski, VT, USA).

### 2.11. Statistical Analysis

Sequence alignments were conducted using DNAMAN Version 10.0 (Lynnon Biosoft, Vaudreuil, Quebec, Canada). ANOVA was performed to compare data from Δ*orf037l*-infected fish with those from the mock-infected ones. Statistical significance was set at a *p*-value of <0.05. Both GraphPad Prism 10.1.2 (GraphPad Software, San Diego, CA, USA) and SPSS 27.0.1 (IBM, Armonk, NY, USA) were employed for the entire data analysis process.

## 3. Results

### 3.1. Construction and Identification of Δorf037l

**Figure 1:**
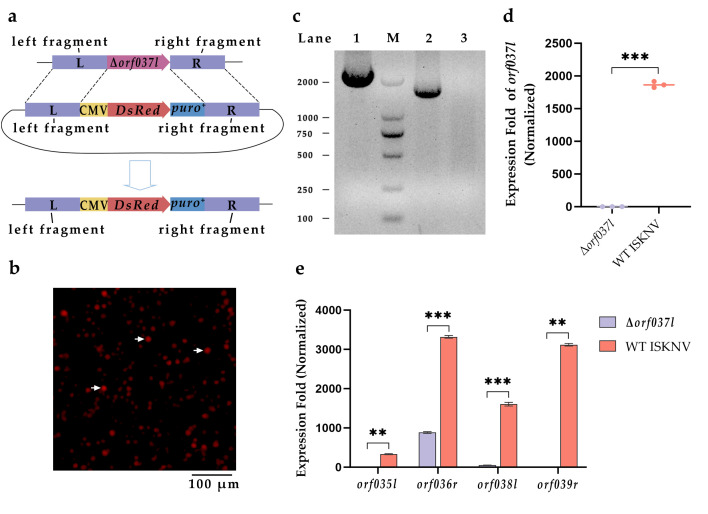
Construction and identification of Δ*orf037l*. (**a**) Schematic diagram showing the construction of the ISKNV *orf037l* recombinant virus. CMV denotes the cytomegalovirus immediate-early promoter. L indicates the upstream arm of the *orf037l*; R indicates the downstream arm of the *orf037l*; *DsRed* indicates the *red fluorescent protein* gene, and *puro^+^* signifies the *puromycin resistance* gene. (**b**) Inversed fluorescent microscope image of MFF-1 cells infected with the *orf037l* recombinant virus. The arrow points to the cells infected by the recombinant virus emitting red fluorescence. Scale bar: 100 µm. (**c**) PCR analysis for assessing the purity of Δ*orf037l* virus. 1: Tested DNA; 2: ISKNV wild-type virus; 3: Negative control; M: DS 2000 marker. (**d**) mRNA expression levels of *orf037l* in infected cells. (**e**) Expression levels of flanking genes in virus-infected cells. Statistical significance is indicated by asterisks, with ** *p*-value < 0.01, and *** *p*-value < 0.001.

Using approximately 1000 bp of sequences flanking the *orf037l* gene, serving as the left and right homology arms, these fragments were integrated with a recombinant transfer vector harboring the *DsRed fluorescent protein* and *puromycin resistance* gene cassette. The ISKNV *orf037l* recombinant transfer vector was then constructed and transfected into MFF-1 cells, subsequently infected with ISKNV to generate a recombinant virus wherein the *orf037l* gene was replaced by the *DsRed* and *puromycin resistance* genes (Figure 1a). Observations conducted through a fluorescent inverted microscope revealed that cells infected with the recombinant emitted a distinct red fluorescence in the affected regions, indicating successful homologous recombination. After undergoing five rounds of puromycin selection, along with multiple separations and purifications of plaques exhibiting red fluorescence, it was observed that all cells infected by the purified virus strains emitted red fluorescence (Figure 1b).

To ascertain the purity of Δ*orf037l*, viral DNA was extracted and subjected to PCR amplification using primers flanking the *orf037l* gene. The results show that the amplified product of the Δ*orf037l* genome consisted of a single band, matching the size of the tagged gene (approximately 2000 bp), whereas the wild-type ISKNV genome yielded a single band of about 1500 bp (Figure 1c). Upon sequencing and comparison, the amplified bands were found to be 100% identical to the tagged gene sequence. Furthermore, RT-qPCR analysis revealed the absence of *orf037l* mRNA expression in Δ*orf037l*-infected cells, contrasting with normal expression in wild-type ISKNV-infected cells (Figure 1d). These results confirm the successful replacement of *orf037l* by the tagged gene within the ISKNV genome and the purification of the recombinant virus for subsequent experiments.

The deletion of *orf037l* may have implications on the expression of flanking genes. Through RT-qPCR analysis, the mRNA expression levels of *orf035l* to *orf039r* were examined in cells infected with both wild-type ISKNV and Δ*orf037l* (Figure 1e). Notably, the *orf035l* and *orf039r* genes were virtually non-expressed in Δ*orf037l*-infected cells. The expression of *orf036r* decreased by about threefold, while *orf038l* expression diminished by roughly 30 times compared to wild-type ISKNV-infected cells. These results indicate that the deletion of *orf037l* gene impacts the expression of neighboring genes from *orf035l* to *orf039r*. In addition, we conducted a comparative analysis of the replication curves for Δ*orf037l* and WT ISKNV in cells, revealing that the replication rate of Δ*orf037l* was marginally faster than that of WT ISKNV; however, no significant order-of-magnitude difference was observed. The comprehensive results are presented in the Supplementary Material Figure S1.

### 3.2. The Challenge of Immersion Immunization with Δorf037l

**Figure 2:**
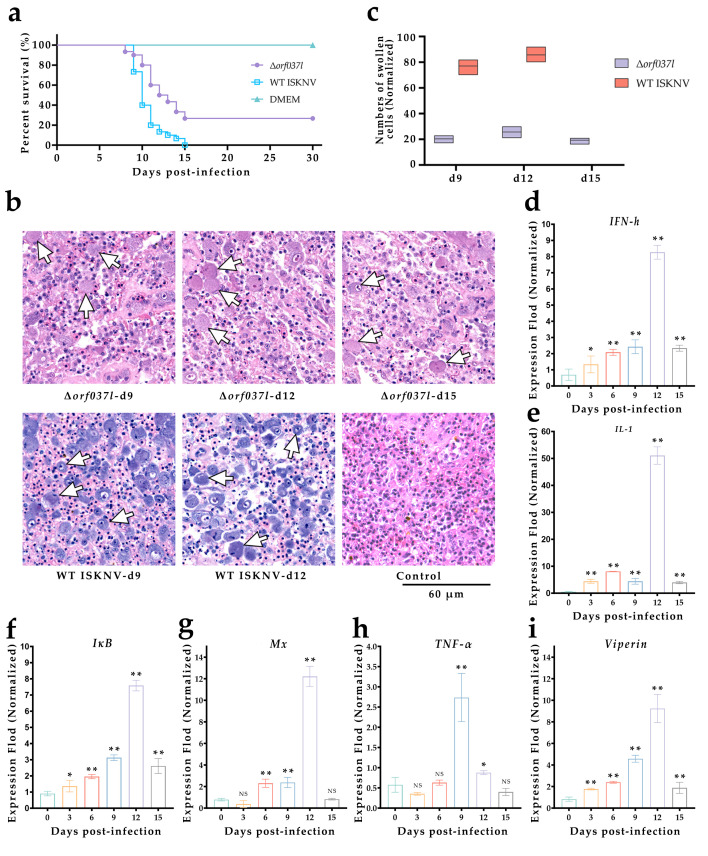
Assessment of the pathogenicity of Δ*orf037l* immersion immunization in mandarin fish. (**a**) Percent survival of mandarin fish infected with Δ*orf037l* at 28 °C. (**b**) Spleen tissue sections of mandarin fish at 28 °C. The arrow indicates the enlarged cells. Scale bar: 60 µm. (**c**) Statistical count of swollen cell numbers under equal visual fields. (**d**–**i**) Changes in the expression levels of *IFN-h*, *IL-1*, *Iκb*, *Mx*, *TNF-α,* and *Viperin* in mandarin fish infected with Δ*orf037l* at 28 °C. Statistical significance is indicated by asterisks, with * *p*-value 0.05 and ** *p*-value 0.01.

In the immunization experiment conducted at 28 °C, each group, comprising 30 mandarin fish, served as the basis for the statistical analysis of survival rates. After immersion infection, the fish were monitored continuously for 30 days, with daily survival rates recorded (Figure 2a). It was observed that the Δ*orf037l* group exhibited a survival rate of 26.7%, whereas the WT group demonstrated 0% survival. Therefore, a preliminarily assessment indicated a diminished pathogenicity of Δ*orf037l* relative to the WT virus.

Furthermore, the sampling group, each containing thirty mandarin fish, underwent immersion infection, with three fish from each experimental group sampled every three days. Notably, no survivors remained in the Δ*orf037l* group after the 15th day of sampling, whereas the WT group was devoid of survivors by the 12th day. Spleens from these fish were dissected for tissue fixation and sectioning, enabling the observation of cellular status (Figure 2b). Spleens from the Δ*orf037l* group exhibited significantly enlarged cells, though this phenomenon was even more pronounced in the WT ISKNV control group. Enumeration of enlarged cells in three randomly selected, equal-sized areas within each section (Figure 2c) revealed that on the 9th and 12th days, the average count was significantly higher in the WT group compared to the Δ*orf037l* group (*p* < 0.01), further substantiating the reduced pathogenicity of Δ*orf037l*.

RNA was extracted from the dissected spleens of the mandarin fish infected or uninfected with Δ*orf037l*, and the relative quantification was performed after reverse transcription, with *β-actin* as the internal reference, to detect the changes in the expression levels of immune-related genes *IFN-h*, *IL-1*, *IκB*, *Mx*, *TNF-α,* and *Viperin* in the fish (Figure 2d–i). The Δ*orf037l* group exhibited the highest upregulation of *IL-1*, followed by *Mx*, whereas *IFN-h*, *IκB*, and *Viperin* displayed similar upregulation patterns, with *TNF-α* upregulation being insignificant. All six immune-related genes demonstrated an initial increase followed by a decrease in expression levels, with the exception of *TNF-α*, which peaked on the 9th day, whereas the remaining genes peaked on the 12th day. The results indicate that Δ*orf037l* effectively induces the innate immune response and possesses robust immunogenicity in mandarin fish.

**Figure 3:**
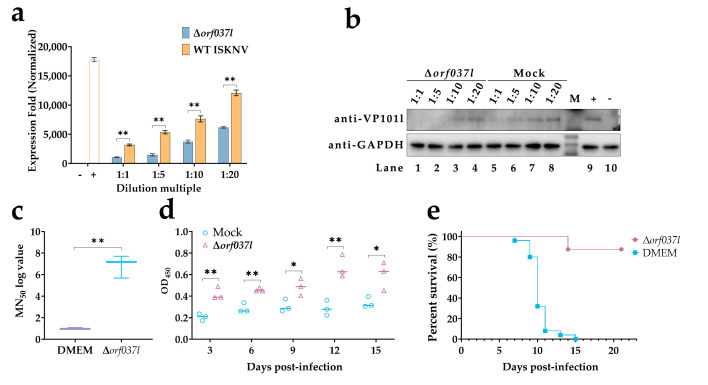
Evaluation of protective efficacy of Δ*orf037l* immersion immunization in mandarin fish. (**a**) RT-qPCR detection of the neutralizing ability of specific antibodies in mandarin fish serum against the virus on day 12 at 28 °C. “−” indicates the blank control group without WT ISKNV and serum, and “+” indicates the positive control group with only WT ISKNV added. (**b**) Western blot detection of the neutralizing ability of specific antibodies in mandarin fish serum against the virus on day 12 at 28 °C. Lanes 1 to 4 are the Δ*orf037l* group; lanes 5 to 8 are the mock group; lane 9 is the positive control group, and lane 10 is the negative control group, where 1, 5, 10, and 20, respectively, represent the four concentration gradients of 1:1, 1:5, 1:10, and 1:20. “+” indicates WT ISKNV only, and “–” indicates no WT ISKNV and serum added. (**c**) MN_50_ titers of the serum samples on the 12th day post-immersion were measured at 28 °C. (**d**) Determination of specific antibody IgM levels in mandarin fish serum at 28 °C. (**e**) Percent survival of mandarin fish under the protection of Δ*orf037l* at 28 °C. Statistical significance is indicated by asterisks, with * *p*-value 0.05 and ** *p*-value 0.01.

To ascertain the ability of Δ*orf037l* to induce host-specific immune responses, mandarin fish serum was collected on the 12th day after immunization from both the Δ*orf037l* and DMEM (mock) groups to detect the neutralizing effect of the serum on ISKNV (Figure 3a). The results indicate that under different dilution ratios, the expression of ISKNV *mcp* was significantly lower in the Δ*orf037l* group compared to the control group, showing a dose-dependent effect. Western blot analysis revealed weaker protein bands in the Δ*orf037l* group at equivalent dilution ratios compared to the control (Figure 3b). These findings confirm that Δ*orf037l* elicits the production of ISKNV-specific neutralizing antibodies in mandarin fish, with the corresponding serum exhibiting neutralizing capabilities against ISKNV. Quantification of ISKNV-specific NAbs in serum (Figure 3c) revealed an MN_50_ titer of 102.14 in the Δ*orf037l* serum, significantly higher than the less than 10 titers observed in the DMEM serum, underscoring the potent induction of ISKNV-specific NAbs by Δ*orf037l* on the 12th day post-immersion. Given the importance of specific IgM antibody activation levels in aquaculture vaccine efficacy evaluation, ELISA was employed to assess IgM levels in mandarin fish serum (Figure 3d). While the control group’s IgM levels remained largely unchanged, Δ*orf037l* serum IgM levels exhibited an initial increase, peaking on the 12th day, and were significantly elevated compared to the control group. These results confirm that Δ*orf037l* elicits the production of ISKNV-specific IgM in mandarin fish.

For the challenge test assessing the protective effect of Δ*orf037l* on mandarin fish survival at 28 °C, observations were extended for 21 days, with daily survival rates recorded (Figure 3e). The Δ*orf037l* group reached a survival rate of 87.5%, whereas all fish in the DMEM group succumbed by the 7th day, resulting in a 0% survival rate. These results indicate that the surviving, immunized mandarin fish in the Δ*orf037l* group produced immune protection against ISKNV, highlighting the strong immune protective effects of Δ*orf037l*.

The above results underscore the favorable immunogenicity and protective efficacy of Δ*orf037l*, which effectively elicits both innate and adaptive immune responses in mandarin fish, thereby being a promising candidate for the development of an ISKNV immersion vaccine. However, the relatively high mortality rate observed in mandarin fish at 28 °C necessitates the exploration of effective strategies to further attenuate the virulence of Δ*orf037l*.

### 3.3. Evaluation of the Protective Efficacy Induced by Low-Temperature Immersion Immunization with Δorf037l

**Figure 4:**
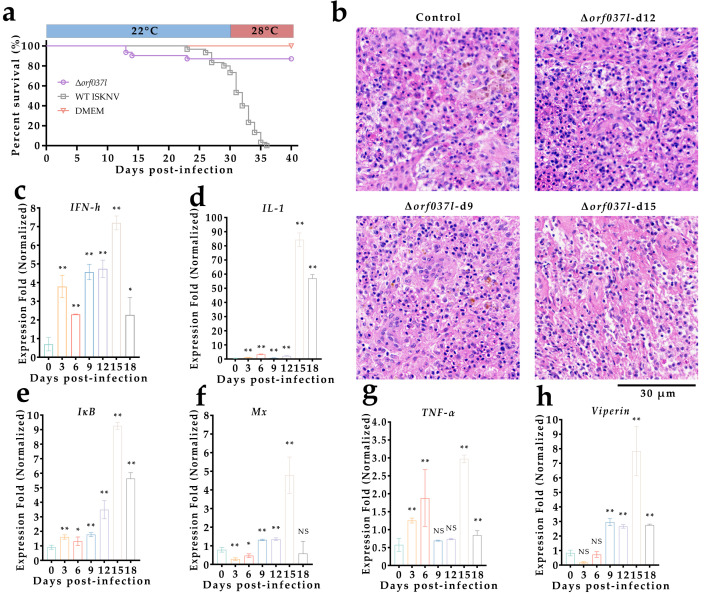
Evaluation of immunological efficacy induced by low-temperature immersion immunization with Δ*orf037l*. (**a**) Percent survival of mandarin fish infected with Δ*orf037l* at 22 °C. (**b**) Spleen tissue sections of mandarin fish at 22 °C. The arrow indicates the enlarged cells. Scale bar: 60 µm. (**c**–**h**) Changes in the expression levels of *IFN-h*, *IL-1*, *IκB*, *Mx*, *TNF-α,* and *Viperin* in mandarin fish infected with Δ*orf037l* at 22 °C. Statistical significance is indicated by asterisks, with * *p*-value 0.05 and ** *p*-value 0.01.

To further mitigate the virulence of Δ*orf037l*, an immunization experiment was conducted at 22 °C, wherein mandarin fish were infected via the same immersion method. Following 30 days of continuous observation, the water temperature was elevated to 28 °C for an additional 10 days, during which the daily survival rates were recorded (Figure 4a). The results show that the Δ*orf037l* group exhibited a survival rate of 90%, whereas the WT group displayed 0% survival. These observations imply that under low-temperature conditions, the virulence of Δ*orf037l* is further diminished and does not lead to the death of mandarin fish upon temperature restoration. In contrast, WT ISKNV prolongs the pathogenic onset in mandarin fish at low temperature but ultimately results in the death of all mandarin fish upon temperature recovery, preliminarily proving the general feasibility of the low-temperature immunization strategy in mitigating the virulence of the gene-deletion virus strains.

Furthermore, in each experiment, a sampling group comprising thirty mandarin fish was established and infected identically. Every three days, three fish from each group were sampled, and their spleens were extracted, fixed, and sectioned for cellular status observation (Figure 4b). No swollen cells were discernible in the spleens of Δ*orf037l*-infected fish, further indicating that the attenuation of Δ*orf037l* virulence at 22 °C.

RNA was extracted from the extracted mandarin fish spleens and reverse-transcribed, and the relative quantification was performed using *β-actin* as an internal reference to detect changes in the expression levels of immune-related genes in mandarin fish (Figure 4c–h). Compared to day 0, the Δ*orf037l* group demonstrated the highest upregulation of *IL-1*, with similar upregulation patterns observed for *IFN-h*, *IκB*, *Mx*, and *Viperin*, whereas *TNF-α* upregulation was insignificant. The expression trend of these immune-related genes indicated an initial increase followed by a decrease, peaking on day 15. This suggests that Δ*orf037l’s* ability to elicit an innate immune response in mandarin fish at low temperature (22 °C), without compromising its immune effect. Moreover, temperature may influence the timing of immune gene expression peaks, which were slightly delayed at lower temperatures.

**Figure 5:**
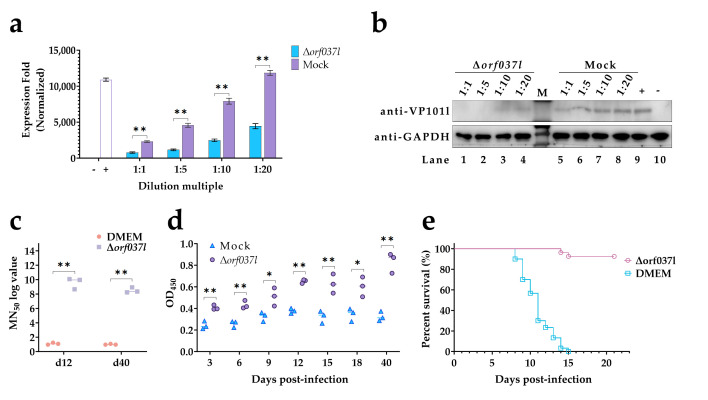
Evaluation of protective efficacy induced by low-temperature immersion immunization with Δ*orf037l* and detection of specific antibodies levels. (**a**) The neutralizing ability of specific antibodies in mandarin fish serum against the virus was assessed on day 12 at 22 °C. After co-incubation with the virus and serum, the virus infected MFF-1 cells, followed by detection of the virus’s *mcp* gene via RT-qPCR. “−” indicates the blank control group without WT ISKNV and serum, and “+” indicates the positive control group with only WT ISKNV added. (**b**) Western blot detection of the neutralizing ability of specific antibodies in mandarin fish serum against the virus on day 12 at 22 °C. Lanes 1 to 4 are the Δ*orf037l* group; lanes 5 to 8 are the mock group; lane 9 is the positive control group, and lane 10 is the negative control group, where 1, 5, 10, and 20, respectively, represent the four concentration gradients of 1:1, 1:5, 1:10, and 1:20. “+” indicates WT ISKNV only, and “–” indicates no WT ISKNV and serum added. (**c**) MN_50_ titers of the serum samples on the 12th and 40th days post-immersion were measured at 22 °C. (**d**) Determination of specific antibody IgM levels in mandarin fish serum at 22 °C. (**e**) Percent survival of mandarin fish under the protection of Δ*orf037l* at 22 °C. Statistical significance is indicated by asterisks, with * *p*-value 0.05 and ** *p*-value 0.01.

On the 12th day post-immunization, serum was collected from both the Δ*orf037l* and DMEM (mock) groups to evaluate the neutralizing abilities against the virus (Figure 5a). The results show that at different dilutions, the expression level of ISKNV *mcp* in the Δ*orf037l* group was significantly lower than in the control group, exhibiting a dose-dependent effect. Western blot analysis confirmed weaker protein bands in the Δ*orf037l* group at equivalent dilutions (Figure 5b). These results indicate that Δ*orf037l* can induce the production of ISKNV-specific NAbs in mandarin fish at low temperature (22 °C). Moreover, neutralizing antibody titers in serum samples collected on the 12th and 40th days were assessed (Figure 5c), revealing ISKNV-specific NAbs titers of 102.99 and 102.66, respectively, in the Δ*orf037l* serum, while control group titers remained below 10 at both time points. This suggests that Δ*orf037l* elicits sustained high levels of ISKNV-specific NAbs over extended periods at low temperature (22 °C). ELISA analysis was performed to detect specific IgM antibody levels in mandarin fish serum (Figure 5d). While control group IgM levels remained largely unchanged post-immersion, Δ*orf037l* serum IgM levels exhibited slight fluctuations but an overall upward trend, maintaining high levels from days 12 to 40, significantly exceeding control group levels. This confirms Δ*orf037l*’s ability to induce the production of sustained high levels of ISKNV-specific IgM antibodies in mandarin fish at low temperature (22 °C). These results underscore the unimpaired immunogenicity of Δ*orf037l* at low temperature (22 °C), capable of eliciting a robust innate and adaptive immune response in mandarin fish. This validates the low-temperature immunization strategy’s feasibility in reducing the virulence of gene-deletion vaccines and highlights Δ*orf037l*’s potential as an effective immersion vaccine against ISKNV.

In a challenge test conducted on mandarin fish surviving the 22 °C immunization, the protective effect of Δ*orf037l* was evaluated over 21 days of continuous observation (Figure 5e). The Δ*orf037l* group achieved a survival rate of 92.6%, whereas the DMEM group experienced complete mortality by day 15. These results demonstrate that Δ*orf037l*’s protective effect in mandarin fish at low temperature (22 °C), achieving a high protection rate with minimal mortality, positions it as a safe and efficient candidate immersion vaccine for ISKNV under low-temperature conditions.

## 4. Discussion

**Figure 6:**
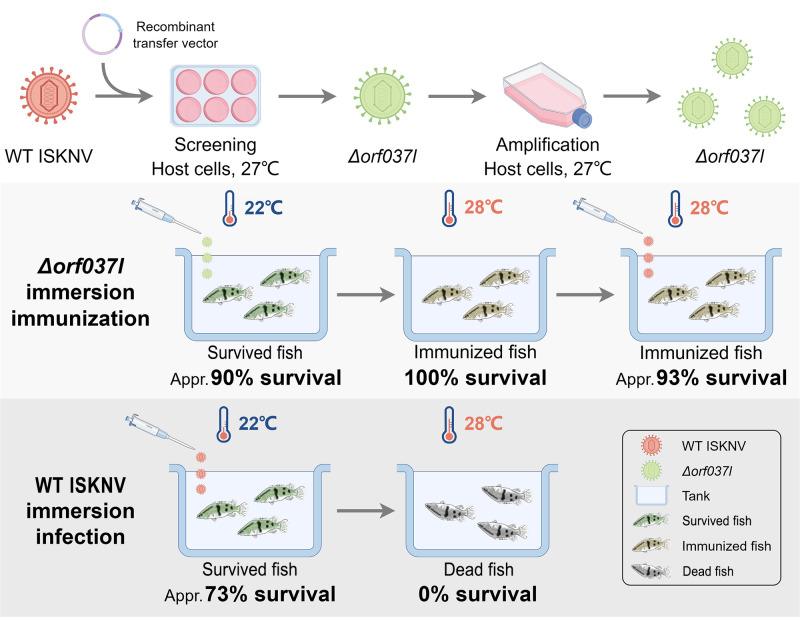
Schematic diagram of the immunization and challenge experiments. Low-temperature immunization reduces the virulence of Δ*orf037l*, improves its immune survival to 90%, and can achieve a RPS of 92.6%.

In this study, a gene-deleted strain, Δ*orf037l*, was generated through the utilization of homologous recombination technology to delete the ISKNV *orf037l* gene. This strain demonstrates a pathogenicity of 73.3% against cultured mandarin fish at a standard temperature of 28 °C, exhibiting a 26.7% decrease in virulence when compared to the WT ISKNV. Based on the temperature optimization scheme depicted in Figure 6, the virulence of Δ*orf037l* further decreased at a low temperature of 22 °C, leading to a mortality rate of only 10% for the cultured mandarin fish. Furthermore, even at 22 °C, Δ*orf037l*, upon being administered via immersion, is capable of stimulating both the innate and adaptive immune responses in mandarin fish. This offers the farmed mandarin fish a remarkable RPS of up to 92.6%, thereby presenting itself as a potential candidate vaccine for low-temperature immersion immunization. Our findings underscore the importance of temperature optimization in the research and application of immersion vaccines, which holds significant implications for advancing the development of immersion vaccines.

Currently, published investigations indicate that ISKNV-like megalocytiviruses possess the capability to infect over 60 species of both farmed and wild fish [5]. Moreover, frequent reports of ISKNV infection events exist [48,49,50,51,52]. Specifically, in China, ISKNV stands as the primary pathogen causing epidemiological outbreaks in cultured mandarin fish (*S. chuatsi*), annually inflicting significant economic losses on the mandarin fish aquaculture industry. Progress has been made in the development of a vaccine against ISKNV, and an ISKNV-specific, fully inactivated vaccine that can be administered by injection has been commercialized in Singapore and China. [26]. Injectable immunization is a very well-established and cost-effective way of administering vaccines, where a small amount of antigen can provide effective immune protection. However, compared to injection immunization, there are some advantages to immersion immunization, such as easy of operation, suitable for fry and juvenile fish, and less stressful reactions. [21]. The development of live-attenuated vaccines that can be administered via immersion holds utmost importance for mandarin fish viral disease prevention and control, along with advancing the aquaculture industry. The gene-deletion attenuated vaccine enables DNA modification without restrictions imposed by nucleic acid endonucleases or DNA ligases. Attenuated strains, created by the knockout of one or more virulence genes, exhibit clear genetic backgrounds, reduced virulence, and decreased pathogenicity [53]. In this study, we successfully knocked out the ISKNV *orf037l* gene, yielding the *orf037l* gene-deletion attenuated vaccine candidate strain, suitable for immersion administration and contributing to the prevention and control of ISKND.

Furthermore, genes exhibiting a high degree of homology to the amino acid sequence of ISKNV *orf037l* have been identified in other *Megalocytivirus pagrus1*, such as OSGIV [45] and large yellow croaker iridovirus (LYCIV) [54]. OSGIV and LYCIV are both categorized as subtypes of RSIV, which is classified under the *Megalocytivirus pagrus1* species. Specifically, OSGIV is assigned to the RSIV-I subtype, whereas LYCIV is designated as belonging to the RSIV-II subtype [4]. One of the genes in LYCIV sharing homology with ISKNV *orf037l* is also named *ofr037l*, encoding a protein believed to be involved in viral infection [54]. This indicates that the orf037l gene may be regarded as a potential target for developing a live-attenuated virus vaccine. Upon the knocking out of the ISKNV *orf037l* gene, it was observed that at 28 °C, the virulence of Δ*orf037l* decreased significantly compared to WT ISKNV, with a 26.7% reduction in mortality rate, indicating that *orf037l* is a virulence-related factor of ISKNV. Additionally, the downregulation of multiple genes may influence the viral replication capacity and virulence within the host; however, further investigation is required to elucidate the precise mechanisms and effects of these alterations. However, Δ*orf037l*, when considered for vaccine application, has a high mortality rate at normal temperatures (28 °C), thereby lacking economic and practical feasibility for application as a vaccine. Recently, our study on another gene-deletion vaccine targeting ISKNV *orf074l* suggests that optimizing the ambient temperature improves the efficacy of the immersion vaccine [34]. This stands as the sole existing research exploring the impact of water temperature optimization on gene-deletion attenuated vaccine efficacy. Inspired by this, our study aimed to further attenuate the virulence of Δ*orf037l* by adjusting the water temperature during immersion, positioning it as a more economically viable vaccine candidate. Moreover, our findings demonstrate the broader applicability of water temperature optimization in providing vaccine efficacy.

Currently, the molecular mechanisms whereby temperature optimization contributes to vaccine application value and reduces vaccine virulence remain underexplored. This is primarily due to the complex effects of temperature variations on both the host and the pathogen. For the host, these effects are multifaceted, presenting both favorable and unfavorable outcomes [55,56]. In the context of viruses, studies have demonstrated that temperature shifts can influence their virulence, potentially linked to the biological characteristics of the pathogen [41,42]. Several studies have proposed that the decreased mortality rates observed at non-lethal virus temperatures may correlate with the activation of the host’s immune activation. For instance, spring viremia of carp virus often leads to high mortality in carp farms, especially during spring when temperatures hover between 10 °C and 17 °C. However, at elevated temperatures, carp develop humoral immunity, offering protection against re-infection [57]. Similarly, in the case of olive flounder (*Paralichthys olivaceus*) infected with hirame novirhabdovirus (HIRRV), temperatures ranging from 10 °C to 20 °C significantly reduce mortality. Prolonged exposure of infected halibut to 20 °C stimulates robust antibody-mediated immunity, protecting against HIRRV re-infection [58]. A recent study on the attenuated vaccine for cyprinid herpesvirus 2 demonstrated that the viral load of the vaccine virus was influenced by temperatures within the range of 15–30 °C; however, its protective efficacy remained intact [59]. As a vaccine, one crucial characteristic is its ability to elicit a potent innate and adaptive immune response in the host. Δ*orf037l*, owing to its high pathogenicity at 28 °C, triggers a host immune response. Notably, even at a low temperature of 22 °C, Δ*orf037l* elicits a robust innate and adaptive immune response. This might explain why, following temperature optimization, the virulence of Δ*orf037l* diminishes, positioning it as a promising vaccine candidate. In order to increase culture production and economic efficiency, aquaculture around the world in recent years has shown a trend toward intensification, i.e., an increase in the proportion of bait species production, the use of intensive aquaculture systems (cage, pens, waterways, and indoor facilities), and the application of oxygenators [60]. Intensive aquaculture is a sustainable method of aquaculture that achieves control over the aquatic environment. It effectively reduces environmental pollution and regulates water quality indicators such as water temperature, pH, and particulate matter levels [61]. The water temperature optimization strategy is very suitable for use in these systems, as intensive aquaculture systems can easily regulate water temperature to the required low temperature for vaccine immersion.

In conclusion, a novel ISKNV gene-deletion candidate immersion vaccine strain, Δ*orf037l*, was developed, achieving a remarkable RPS of 92.6% in mandarin fish via low-temperature immersion (22 °C). Moreover, the significance of optimizing ambient temperature during the immunization procedure of an immersion vaccine is further emphasized, highlighting its potential to elevate the value of the vaccine.

## 5. Patents

An application for a national invention patent of the PR China (no. 2024064662300) was submitted for Δ*orf037l*.

## Figures and Tables

**Table 1:** Primers in this study.

Name	Experiment	Sequences (from 5′ to 3′)
*orf037l*-left-F	PCR	CCCAAGCTTTGCCACATCCATCCTTACAG
*orf037l*-left-R	PCR	CGGGATCCCGGAAAAGATGATAGGCGTG
*orf037l*-right-F	PCR	GGGGTACCAGTCAGTTTTATATCATCAA
*orf037l*-right-R	PCR	CGGAATTCGTTGACCTGGGTTCAGGCCA
*orf037l*-outer-F	PCR	CCCACCGCCACATTGCCAAGT
*orf037l*-outer-R	PCR	CCCCGGCCAAAAAGATATACAACG
isknv*orf037l*-qF	RT-qPCR	AGACACGAGCCACAAACTGT
isknv*orf037l*-qR	RT-qPCR	CTCACGCAGCTTATTGACGC
isknv*mcp*-qF	RT-qPCR	CAATGTAGCACCCGCACTGACC
isknv*mcp*-qR	RT-qPCR	ACCTCACGCTCCTCACTTGTC
*scβ-actin*-qF	RT-qPCR	CCCTCTGAACCCCAAAGCCA
*scβ-actin*-qR	RT-qPCR	CAGCCTGGATGGCAACGTACA
isknv*orf035l*-qF	RT-qPCR	CGTACTTGTGCAACACTGCC
isknv*orf035l*-qR	RT-qPCR	GTGGCCATGTATACCGAGGG
isknv*orf036r*-qF	RT-qPCR	TTACTGCGTGGAACGAGTCC
isknv*orf036r*-qR	RT-qPCR	TTGGCACGGAATGCCTGTAT
isknv*orf038l*-qF	RT-qPCR	CATGGTGACAGTCGAAGGCT
isknv*orf038l*-qR	RT-qPCR	CGAATGGCGTGAGGGTATGA
isknv*orf039r*-qF	RT-qPCR	CCGCAAACCTTTGATGCCAA
isknv*orf039r*-qR	RT-qPCR	GAAATGGCGCATAGCCACAG
*scIFN-h*-qF	RT-qPCR	CGCTCTGCTGTGATTGGC
*scIFN-h*-qR	RT-qPCR	GGGACTCCACCTCTGCCTTT
*scMx-qF*	RT-qPCR	GGATTCTGACATCGGGAGCAA
*scMx-qR*	RT-qPCR	GTGCAGTAGACTCATGCTGT
*scIκb-qF*	RT-qPCR	CAGACATCAACGCACAGGAA
*scIκb-qR*	RT-qPCR	CGTGAAGCCGCCATAGTTAA
*scVIPERIN-qF*	RT-qPCR	CCAAGAGGGGCCTCAAACTT
*scVIPERIN-qR*	RT-qPCR	CTGACACTTGGGAGCTGGAG
*scIL-1-qF*	RT-qPCR	GGACAGCGACATGGTGCGATT
*scIL-1-qR*	RT-qPCR	TTGAAGGTTCGGTGGCGTTGG
*scTNF-α-qF*	RT-qPCR	AGCCAGGCATCGTTCAGAGTCT
*scTNF-α-qR*	RT-qPCR	CTGTCCTCCTGAGCGGTGTCTT

## Data Availability

The authors confirm that the data supporting the findings of this study are available within the article and its Supplementary Materials.

## Supplementary Materials

The following supporting information can be downloaded at https://www.mdpi.com/article/10.3390/vaccines12101170/s1, Figure S1: Comparison of replication curves of Δ*orf037l* and WT ISKNV.
