# Supplementary material for: Evaluation of a Low-Temperature Immersion Immunization Strategy for the Infectious Spleen and Kidney Necrosis Virus orf037l Gene-Deleted Attenuated Vaccine
Source: Vaccines (Basel). 2024 Oct 14;12(10):1170. doi: 10.3390/vaccines12101170 (PMC11511343; doi:10.3390/vaccines12101170)
Supplement: Supplementary file 1 [file vaccines-12-01170-s001.zip › vaccines-3225447-supplementary.pdf]

Supplementary Materials

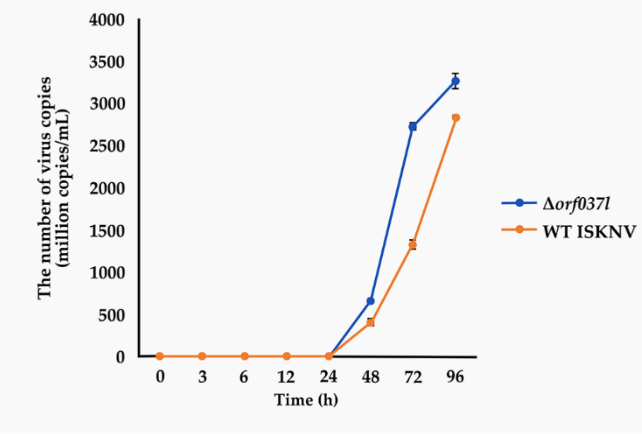

Figure S1. Comparison of replication curves of Δ*orf037l* and WT ISKNV. Equal amounts of Δ*orf037l* and WT ISKNV were infected into MFF-1 cells, and cell samples were collected at 0, 3, 6, 12, 24, 48, 72, and 96 h post-infection, and DNA was extracted for absolute quantification, and the replication curves of Δ*or037l* and WT ISKNV were plotted.
